# Supplementary material for: Smoking and pre-existing co-morbidities as risk factors for developing severity of COVID-19 infection: Evidence from a field hospital in a rural area of Bangladesh
Source: PLoS One. 2023 Dec 8;18(12):e0295040. doi: 10.1371/journal.pone.0295040 (PMC10707513; doi:10.1371/journal.pone.0295040)
Supplement: Attachment — Submitted filename: To Authors.docx [file pone.0295040.s002.docx]

Please clearly mentioned the sampling procedures and the population and calculation of sample in Methodology section. Give very early and current study synchronization in Literature review section. Clearly state the disease severity and patient’s characteristics and properly acknowledge it in Result section Compare and contrast with the current available evidence.

Thanks for your hard work.
