# Supplementary material for: Smoking and pre-existing co-morbidities as risk factors for developing severity of COVID-19 infection: Evidence from a field hospital in a rural area of Bangladesh
Source: PLoS One. 2023 Dec 8;18(12):e0295040. doi: 10.1371/journal.pone.0295040 (PMC10707513; doi:10.1371/journal.pone.0295040)
Supplement: Attachment — Submitted filename: Response of Reviewers.docx [file pone.0295040.s003.docx]

**Reviewer’s Feedback:** Please clearly mentioned the sampling procedures and the population and calculation of sample in Methodology section. Give very early and current study synchronization in Literature review section. Clearly state the disease severity and patient’s characteristics and properly acknowledge it in Result section. Compare and contrast with the current available evidence.

Thanks for your hard work.

**Responses:**

**Sampling procedures and the population:** We explained clearly the sampling procedures and the study population in the “Study design and data source**” in Materials and methods** section.

**Sample size calculation:** According to the inclusion and exclusion criteria, this study included all the patients who were COVID-19 positive and admitted at the studied hospital. This is a retrospective analysis of medical records of the patients. Therefore, the calculation of sample size could be considered as a waive.

For example; the below listed studies also did not consider sample size calculation in their retrospective methodology.

1. Adrish M, Chilimuri S, Mantri N, et al. Association of smoking status with outcomes in hospitalised patients with COVID-19. BMJ Open Resp Res 2020;7:e000716. doi:10.1136/ bmjresp-2020-000716; <https://bmjopenrespres.bmj.com/content/bmjresp/7/1/e000716.full.pdf>

# 2. Asthma and COPD Are Not Risk Factors for ICU Stay and Death in Case of SARS-CoV2 Infection; <https://doi.org/10.1016/j.jaip.2020.09.044>

3. Clinical Characteristics of Coronavirus Disease 2019 in China, N Engl J Med 2020;382:1708-20.DOI: 10.1056/NEJMoa2002032; <https://www.nejm.org/doi/10.1056/nejmoa2002032>

**Other changes:**

- I have now added the following k**eywords:** Severity of COVID-19; SARS-CoV2; Risk factors; Smoking history; Co-morbidities; icddr,b; Bangladesh;” just after the abstract section (line-46).
- I have rephrased the “**Introduction”** to ***“Background”*** (line-48), **“COVID-19 disease severity”** and **“severity of COVID-19 disease”** to ***“severity of COVID-19 infection”*** throughout the manuscript.
- Literature review section is now up-to-date with the clear and current study synchronization. Below are the changes in reference list:
- ***In the pandemic (line 49):*** *Ref. #4 is now moved to the ref. # 1 and ref. #3 move to the ref. #5*; *ref. #2, #3, and #4 is newly inserted;*
- ***In Literature review (line 58)*:** *Ref. #5, #1, #11, #12, #7,* #30, #16, #17, #18, #19, #21, #22, & #6 *is now moved to ref. #6, #7, #10, #11, #12,* #17, #18, #19, #20, #21, #22, #23, & #24 *respectively. Ref. #14 & #16 is newly inserted;*
- ***In Study design and data source (line 105):*** *Ref.* #23 *is now moved to the ref.* #25
- ***In Severity of COVID-19 infection (line 121):*** *Ref.* #25 *is now moved to the ref.* #26
- ***In Statistical analysis (line142):*** *Ref.* #26 *is now moved to the ref.* #27*,* *Ref. #28 is newly inserted;*
- ***In Discussion (line 245):*** *Ref.* #28*,* #29*,* #27*,* #31*,* #32*,* #33, #34*,* #35*, &* #36 *is now moved to ref.* #29*,* #30*,* #31*,* #32*,* #33*,* #34*,* #35*,* #36*, &* #37 *respectively.*
- Disease severity is clearly defined based on the ***“Living Guidance for clinical management of COVID-19 2021 by WHO (line 121 - 138)”.*** Now, the disease severity and patient’s characteristics are clearly stated and properly acknowledged in the **Result section.** Below are the changes:
- In the discussion section, I compared and contrasted with the current available evidences. I made the following changes: “In 2020, a research conducted by the Center for Disease Control (CDC) COVID-19 Response Team in USA showed that smokers had higher risk of having critical condition than never-smoker” by ***“For assessing the impact of smoking on COVID-19 severity, a meta-analysis found that patients who were current or former smoker had an increased risk of severe or critical COVID-19 infection”****.* I have also updated the references in the main text (line 260 - 264). I have also added the paragraph” “We found that CVD are independently associated with severity of COVID-19 infection, which is found to be insignificant when adjusting with its covariates. Similar findings were observed in, Kunihiro et al. study that CVD were independently associated with severity of COVID-19” (line 286 - 289).
- Laboratory protocols is not applicable because the samples are collected in the SARI ITC icddr,b and tested at RT PCR laboratory set-up by World Health Organization in Cox’s Bazar as COVID-19 emergency response and is permitted to use for research purpose.
