# Supplementary material for: Smoking and pre-existing co-morbidities as risk factors for developing severity of COVID-19 infection: Evidence from a field hospital in a rural area of Bangladesh
Source: PLoS One. 2023 Dec 8;18(12):e0295040. doi: 10.1371/journal.pone.0295040 (PMC10707513; doi:10.1371/journal.pone.0295040)
Supplement: Attachment — Submitted filename: Response to Reviewers.docx [file pone.0295040.s004.docx]

Date: 11.08.2023

Subject: Response to Reviewers - Manuscript Number PONE-D-22-33469R1.

Dear Mosharop Hossian,

We would like to express our sincere gratitude to you and the reviewers for taking the time to assess our manuscript titled "**Smoking and Pre-existing Co-morbidities as Risk Factors for Developing Severity of COVID-19 Infection: Evidence from a Field Hospital in a Rural Area of Bangladesh.**" We value the constructive feedback provided by the reviewers, and we have carefully addressed each point raised in the reviews.

Please find our detailed responses below, along with the corresponding changes made in the revised manuscript.

**Reviewer #1:** The Manuscript investigates an interesting topic however; the authors did not provide highly considerable findings as follows:

**Comment 1**- The authors did not get any permission from the patients to use their data. Even if the data are de-identified in the hospital archives, patients shall be asked to get their permission as a part of the ethical rights of patients.

**Response:** Thank you for your feedback. In the ethical clearance section, we mention that the participant provided verbal consent. In order to make it more understandable, we have rephrased it.

**Comment:** 2- The authors did not explain the reason behind applying two different statistical tests for association.

**Response:** We appreciate your comment regarding the statistical tests. The decision to apply two different statistical tests, Chi-square test and Fisher's exact test, for association was based on established statistical principles. While the Chi-square test is commonly used for identifying associations between categorical variables, it may yield less reliable results when the Expected cell frequency is less than 5. In such cases, Fisher's exact test provides a more robust measure. In this study, we found that the Expected cell frequency was less than 5 for certain variables, including Age, Diabetes, COPD, CVD, and Asthma. Hence, we opted for Fisher's exact test to ensure a more accurate and reliable analysis for these specific variables.

**Comment:** 3- The age variable is not investigated properly. The association of age groups with respect to COVID severity shall be used according to the WHO classification of age groups by infection (not below 60 and above 60 years old).

**Response**: Thank you for your valuable feedback. We acknowledge the importance of using age groups based on the WHO classification for COVID-19 severity analysis. Regarding the age variable, we chose to categorize patients as "adults" (18-60 years) and "elderly" (>60 years) due to the observed significant association between these age groups and the severity of COVID-19 infection in the pilot analysis.

**Comment:** 4- The discussion section needs an additional improvement to link the finding of the paper with the published data. The authors compared their findings only with a limited number of published studies. Thus, the contradiction of association (mentioned in the introduction) is not addressed again in the discussion to fill this research gap.

**Response**: Thank you for your suggestions. Based on your suggestions, we have now enhanced the discussion section by adding a few references addressing the contradiction of association.

**Reviewer #2:** Thank you very much for the great work on this manuscript. Just a few comments:

**Comment**: In line 74: “However, patients with ….” Do you mean “However, in other studies patients with.”? if not please rephrase.

**Response:** We appreciate the comment. We have rephrased the sentence in line 74 to clarify the statement with a proper citation which now can be found on line number 98 to 100: " However, a study conducted on symptomatic patients found that COPD and asthma alone did not lead to worsening infections, hospitalizations, or deaths associated with COVID-19 [30]

**Comment:** In line 78: it states there are only a couple of studies in Bangladesh as of now The mentioned one in the manuscript is referenced as [6]: Mohsin FM, Tonmon TT, Nahrin R, Tithy SA, Ame FA, Ara I, et al. Association Between 352 Smoking and COVID-19 Severity: Evidence from Bangladesh. J Multidiscip Healthc 353 [Internet]. 2021 Jul [cited 2021 Aug 26];14:1923. Available from: /pmc/articles/PMC8315768/ What is the other study?

**Response**: We appreciate your keen observation and valuable input. Since we have only found one study as of now, we have rephrased the statement in line 78, which can be found in lines 102 to 104, as follows: " Despite several studies in many countries, to date, only one study has been found in Bangladesh on the associated risk factors for severe COVID-19 infection, such as smoking [6] and other co-morbidities."

**Comment:** Line 85 and 86: can you rephrase this part? the purpose of the hospital was to manage COVID-19 outbreak from when to when?

**Response**: We appreciate the reviewer's comment and helpful suggestion for rephrasing. The revised version of the sentence, now found on line number 92, reads: "As part of the COVID-19 outbreak management, operations began on August 27, 2020, and are still in progress"

**Comment:** Line 90: “…during the study period.” Only during that time? Line 92: “…supplied as well (by the donor)” and line 94: “…deployed to run ….. ensured ...” by who ? or rephrase please.

**Response**: Thank you so much, I really appreciate it. The free of charge is not only during the study period but still in progress. The materials were supplied by the Donor and deployed by the ICDDR, B. The following points are now clearly stated to make it clearer, as shown in line numbers 116-121.

**Comment:** 172: Severity “of”, 334: Conclusion can you please add specific recommendations for smoking and COVID-19 Thank you for the great work again.

**Response**: Thank you for pointing this out. The missing preposition "of" has now been added to new line 191 and the formatting mark has been removed from your mentioned line number, which is now line 370. We have also added the following specific recommendations which can be found in lines number 378 and 384, read as: “It is recommended that public health interventions should prioritize smoking cessation programs and awareness campaigns to reduce smoking prevalence among the population, particularly those with comorbidities. Furthermore, efforts should be made to enhance the early detection and management of hypertension, diabetes, COPD, and asthma in the community to mitigate the severity of COVID-19 infections and improve overall health outcomes.”

**Reviewer #3, Comment:** The manuscript requires improvement in its English language usage. While the content is satisfactory, the writing needs to be enhanced. The author should focus on refining the methods, results, and conclusion sections.

**Response**: Thank you for acknowledging the satisfactory content of our manuscript. We have taken your comments seriously and have focused on refining the English language usage in the methods, results, and conclusion sections to ensure better readability and clarity. We believe that these improvements will enhance the overall presentation of our findings and make them more accessible to the readers. Your valuable input has been instrumental in improving the quality of our manuscript, and we are grateful for your guidance.

**Reviewer #4:** General comments: The authors present a paper on hospital-level data of COVID-19 patients, their disease severity and the association of disease severity and selected risk factors. The study is carefully thought out and statistically sound. There are a few points that the authors might consider to strengthen the overall contribution.

**Comment**: The title of this paper suggests that the primary association that is under investigation is between COVID-19 disease severity and smoking. However, there is a little background on tobacco use behaviors in the country, and the level of detailed data collected and analyzed is very superficial.

**Response**: Thank you for your feedback. We have enhanced our background by adding the following details “Bangladesh is significant, with nearly 35% of adults smoking or smokeless tobacco currently. Since 2004, this lower middle-income country has witnessed a dramatic increase in the health and economic burden linked to tobacco use, as evidenced by primary data from a nationally representative survey of patients with tobacco-related illnesses. Tobacco smoking significantly contributes to noncommunicable diseases (NCDs), including cardiovascular and respiratory conditions, underscoring the imperative to address tobacco control measures to mitigate its impact on both public health and economic growth.”

**Comment**: A stronger rationale for the co-morbidities chosen would help frame the aims of the paper.

**Response**: Rational about some co-morbidities have already been addressed, and the rationale about diabetes as a co-morbidity has been newly added and can be read as” Diabetic patients with COVID-19 have a more severe condition and poorer clinical outcomes, including a higher mortality rate and a higher ICU admission rate, highlighting the necessity for better management strategies in order to improve their prognosis.”

**Comment:** Careful proofreading of the flow of English would help with clarity in some parts (eg – Introduction) of the paper. Also, please check spelling of words.

**Response**: Thank you for your feedback. We have now carefully proofread the manuscript and corrected any misspelled words found.

**Comment:** Introduction: General comments: The authors present a succinct literature review stating the existing research on COVID-19 risk and associated risk factors. It may help to present some background data on the impact of COVID-19 in the country or region. Also, what is the burden of disease and associated risk factors for CVD and diabetes? It would help to have more on this to frame the issue.

**Response**: Thank you for your feedback. We have background data on the impact of COVID-19 in the country (Bangladesh), and this can be found in line # as “. In Bangladesh, the first 3 COVID-19 cases were detected on March 2020[4]and more than 2.0 million COVID-19 cases were identified, including about 29,300 deaths till August 10, 2022”. Although the burden of the co-morbidities like CVD and Diabetes were not our primary focus of the study, however, we have added a background related to burden of diabetes in the country in the introduction section.

**Comment:** Regarding the use of tobacco, what is the most commonly used form of tobacco? If you have data on the frequency or amount smoked per day that would be interesting. Did you account for chewing tobacco use?

**Response**: Thank you for your question. All the patient included in the study verbally acknowledged that they were smoking cigarette. The study did not specifically collect data on the frequency or amount smoked per day. Additionally, chewing tobacco use was not accounted for in the analysis, as the study focused primarily on the association between smoking and COVID-19 severity. However, we have included a clarification in the manuscript to explicitly mention that the study focused on cigarette smoking.

**Comment:** Please add citation for statement on line59: “Smokers are 34% more likely to contract the common flu than non-smokers.”

**Response**: Thank you for pointing this out. We have now added the citation and can be found on the new line number # 60

**Comment:** Methods: Did you have a pre-specified analysis plan? if so it would be good to state this. It is not fully clear from your description of your data, or statistical analysis what “factors” you are assessing other than smoking. What are the other co-morbidities and how were they defined (lab values, cut-offs, self-report)?

**Response**: Thank you for your question. The following were considered in the Analysis plan: “ COPD, hypertension, cardiovascular disease, and diabetes were diagnosed based on patient history, clinical features, and radiological and laboratory findings. The severity of COVID-19 infection was determined based on WHO standards. Descriptive statistics were used to determine the frequency and percentage of background, characteristics, severity levels of COVID-19 infections, pre-existing comorbidities, and smoking history. To assess the association between main variables of interest (e.g. history of smoking, underlying co-morbidities) and severity of COVID-19 infection, Pearson's Chi-square test and Fisher's exact test are used. Multinomial logistic regression models were used to identify potential risk factors and determine the effect size of these factors on the progression of COVID-19 infection severity in the patients. To measure statistical significance, a P-value of 0.05 was considered.”

**Comment**: Results: The results of this study are not surprising. Did you consider any interaction effects (smoking*CVD or smoking*COPD or Diabetes*CVD)?

**Reponse**: Thank you for your question. The study primarily focused on assessing the individual effects of various risk factors on the severity of COVID-19 infection. While the potential for interaction effects, such as smoking interacting with CVD or COPD, and diabetes interacting with CVD, could provide valuable insights, these specific interaction effects were not investigated in this study. The focus was primarily on understanding the independent contributions of each factor to the severity of COVID-19 infection.

Discussion and Conclusion

**Comment:** The data was collected over several waves, and so I wondered if there was any discussion on how vaccination uptake may/may not have moderated the severity of disease? If this was not collected or considered in the analysis, then it should be discussed as a limitation. I think that the discussion could be expanded a bit to draw a connection to the policy and practice implications stated in the conclusion.

**Response***:* Thank you for your insightful feedback. As a result of your feedback, we have mentioned our limitation as 'The vaccination uptake and its potential role in moderating the severity of COVID-19 infection were not considered in the analysis'. Also, the clinical team did not perform spirometry to diagnose COPD.”

Furthermore, to address your point, we have expanded our conclusion and it reads as follows: Also, it is recommended that public health interventions should prioritize smoking cessation programs and awareness campaigns to reduce smoking prevalence among the population, particularly those with comorbidities. Among the vulnerable group of people, the implementation of mass awareness campaigns along with diagnostic infrastructure concerning pre-existing co-morbidities may result in a reduction of severity and mortality rate.”

**Reviewer #5:** Reviewer Comments for "Smoking and Pre-existing Co-morbidities as Risk Factors for Developing Severity of

COVID-19 Infection: Evidence from a Field Hospital in a Rural Area of Bangladesh"

Summary

This study is a retrospective analysis of COVID-19 hospital admissions to a rural hospital in Bangladesh. It is unique in its description of its target population in a rural, low-resource setting. It is commendable to find detailed hospital records that could test the association between co-morbidities like COPD, Hypertension, Asthma and CVD with the propensity to develop moderate, severe and critical COVID-19 infection. It also tested the association between demographic characteristics like age, gender and risk habits like smoking with COVID-19 severity. The authors successfully tested the relationship using bivariate analysis (Chi-square analysis and Fisher's exact test) and multivariate analysis (Multinomial logistic regression).

The results were presented in clear and concise tables. This study is precise in its aims and has successfully met them. The following comments could improve the study's reporting and enhance the paper's quality.

**Abstract**

**Comment:** 1. Line # 23: "Based on inclusion criteria;" seems redundant. You could say that "788 admitted patients were included in the analysis."

**Response**: We appreciate your suggestion. Now, we have made this change and can be found in line number #137

**Comment:** 2. Line # 34: A measure of association to the result would increase its credibility –"It was evident that the patients with at least one of the selected co-morbidities (e.g., hypertension, diabetes, COPD, CVD, asthma) had suffered severe illness of COVID-19 compared to the patients who had no co-morbidity."

**Response**: Thank you so much. We have made the necessary adjustment to the sentence you mentioned: "It was evident that patients with at least one of the selected co-morbidities (such as hypertension, diabetes, COPD, CVD, asthma) exhibited a significantly higher likelihood of experiencing severe illness due to COVID-19 compared to patients without any co-morbidity." This revision emphasizes the statistically significant association between the presence of co-morbidities and the severity of COVID-19 infection, thus strengthening the credibility of our results. You can find this on new line numbers 37-41

Background

The background clearly describes COVID-19 infection and how co-morbidities influence it.

**Materials and Methods**

**Comment:** 1. Line 85-96: The study setting gives a detailed description of the field hospital in Teknaf. A brief description of the catchment area/patient population would enhance this description. One or two lines on the patient population - regarding their socio-economic status, dominant occupation, etc.- will help set the study context.

**Response**: We greatly appreciate your feedback and suggestion to provide more context regarding the catchment area and patient population. While we acknowledge the importance of socio-economic status and its potential impact on the study findings, we regret to inform you that we did not include participants' socio-economic status in our analysis due to the emergency of Covid-19 pandemic at hospital setup. As you correctly pointed out, our analysis primarily focused on factors such as age, sex, smoking history, and underlying co-morbidities in relation to the severity of COVID-19 infection which are considered highly relevant to the COVID-19 severity.

**Comment:** 2. Line 153-154: To improve the multinomial logistic regression model reporting, you could mention the independent variables (predictors) used in the analysis. To be more specific, listing the control variables used in the model that examines the "adjusted relationship" between the severity of COVID-19 infection and smoking could help understand the regression model.

**Response**: Thank you for your valuable feedback. We have addressed your point and rephrased the sentence by adding the control variables and can be found in new line numbers #184-#188 as” The following equation (2) was estimated to examine the relationship between the severity of COVID-19 infection and the history of smoking with control variables, such as age, sex, hypertension, diabetes, COPD, CVD, and asthma.”

**Comment:** 3. Line 141: If a categorical variable like the Severity of Covid-19 infection is used as the dependent variable, it would help to mention the reference category and why it was used as a reference category. The justification could be linked to the implications of the result if possible.

**Response**: Multinomial logistic regression models [27] were used to identify the potential risk factors and measure the effect size of these factors on the progression of severity of COVID-19 infection in the patients where "mild case" was used as a reference category’ because severity increases from mild to critical when the factors of interest are present. We have now rephrased and updated the manuscript.

**Comment:** 4. Line 154: You could include measures of model fit to assess how well the model fits the data. Common goodness-of-fit statistics for multinomial logistic regression include the likelihood ratio chi-square test, the McFadden's pseudo R-squared, or the AIC (Akaike Information Criterion). It is mentioned in the results section but can be mentioned in the methodology section.

**Response**: The following equation (2) was estimated to examine the adjusted relationship between the severity of COVID-19 infection and the history of smoking with control variables. We mentioned the likelihood ratio chi-square test and McFadden's pseudo-R-squared values in the results section for assessing the fit of the model to the data.

**Comment:** 5. Could you justify why current and past smokers were combined as one variable? Was it because of uneven distribution across the two categories? Or was it because of a lack of differentiation between the two categories in the database?

**Response**: Thank you for bringing this to our attention. Our primary focus was to assess the impact of smoking on the severity of COVID-19, rather than evaluating its duration. While there was a distinction between the two categories in our database, the differentiation was based on verbal acknowledgment from the patients. Collecting concrete evidence regarding a patient's smoking status, particularly in the context of the COVID-19 situation, is challenging and often relies on verbal consent due to the limitations posed by the circumstances.

**Comment:** 6. Were there any missing data or incomplete records?

**Response**: Thank you. No missing data or incomplete records were included in the study. We extracted data that aligned with our predefined inclusion criteria, ensuring a comprehensive and complete dataset for our analysis.

Results

**Comment:** 1. Line 170: The title of the table could be more elaborate. You could detail what kind of characteristics-demographic/ clinical you are showing. In addition, you could specify what kind of patients you are describing-COVID-19 patients admitted to the field hospital. A detailed title will help quick readers skim through your results.

**Response**: Thank you for your valuable feedback. We appreciate your suggestion for a more informative and comprehensive title for the table. We have updated the title as per your recommendation to enhance clarity and aid quick readers in comprehending the content. The revised title now reads “Table 1: Distribution of Patient Characteristics and Co-morbidities in COVID-19 Cases”. We hope this revised title effectively communicates the scope of the table's contents and provides a clear overview for readers.

**Comment:** 2. Line 179: Table 2 shows the distribution of variables in addition to association. Could you highlight that in your title?

**Response**: Thank you for your valuable input. We have revised the title of the table to accurately reflect its contents. The new title is: “Table 2: Distribution and Association of Patient Characteristics with Severity of COVID-19 Infection” which highlights both the distribution of patient characteristics and their association with the severity of COVID-19 infection, as indicated in the table.

**Comment:** 3. Line 205: You could list what the other variables/confounders were added to your final model.

**Response**: Thank you for pointing out this. We have now added the other confounders included in the final model and rephrased them as follows” As potential risk factors, the following variables were incorporated into the final model (adjusted model): smoking history, hypertension, diabetes, COPD, CVD, asthma, gender, and age (>60 years old - elderly). In the adjusted model, while smoking history was recognized as a risk factor contributing to the severity of COVID-19 infection, it was not associated with moderate and critical levels of severity.” You can find this on the new line number # 246-251

**Comment:** 4. Line 207-271: Please rework reporting the RRR interpretation. For example, in Line 208: It would make sense if you could say that. The outcome and the comparator group must be reported together. The different categories must not be compared with each other unless the analysis puts one of them as the reference category.

5. Line 217: Please specify the comparator group(those not suffering from COPD).

6. Line 218: Please specify the comparator group similar to how you have described in line 224.

7. Line 223: RRR shows increased odds of developing moderate/severe/critical COVID-19 infection from the reference mild COVID-19 infection. You could report … "more risks higher odds of reporting critical, severe, and moderate COVID-19 infected patients, infections than mild COVID-19 infection, when compared to adults …Reference category has to be specified when interpreting the relative risk ratio.

**Responses (4, 5, 6, 7):** Thank you very much for your valuable suggestion. The sentences have now been rephrased based on your feedback and can be found on new lines 250- 270

Discussion

**Comment:** 1. Line 303: Can you rule out misclassification errors that could occur if the patient did not know if they had CVD, COPD or hypertension prior to COVID-19 infection? Were they tested for all these co-morbidities in the hospital? Were these comorbidities diagnosed prior to the admission (hospital records of the only facility in the area) or during admission (first time patients, refugees with no prior records) etc.

**Response**: Thank you for your feedback. While the patients in our study were tested clinically and laboratory confirmation for these co-morbidities during their hospital stay, there is a possibility that some patients may not have been aware of their pre-existing conditions. This particularly applies to first-time patients, like the Forcibly Displaced Myanmar Nationals (FDMN) refugees, who might lack prior medical records. These points have also been addressed and rephrased in the manuscript and can be found on lines 138-140 in the methodology section.

**Comment:** 2. If you could describe your study population, the results could be generalized to this group. This could be a strength of your study, as it describes the particular group.

**Response**: We appreciate the idea. This study population can be found in the study setting section, which states that "The hospital is a not-for-profit organization serving locals, and Forcibly Displaced Myanmar Nationals (FDMN) in Teknaf."

**Comment:** 3. Line 311: As you mentioned that a significant proportion of the population is unaware of their co-morbidities, you could suggest mass awareness campaigns should be coupled with diagnostic infrastructure the target population could access.

**Response**: Thank you for sharing your thoughts. Our conclusion section now discusses the need to link mass awareness campaigns with diagnostic infrastructure and can be found on line 378-380.

Acknowledgment

**Comment:** I am glad that the authors have acknowledged the support staff and managers who make hospital records a rich resource for researchers. To be able to extract data without missing reports or incomplete reports is a feat.

**Response**: Yes, acknowledging the staff behind the data is an honor and we appreciate your valuable comments.

In addition to addressing the specific points raised by the reviewers, we have carefully proofread and improved the manuscript language to correct any grammatical errors and ensure clarity. We believe that these revisions significantly enhance the quality and impact of the manuscript.

Once again, we are grateful for the opportunity to revise and resubmit our work. We sincerely hope that the changes made in the manuscript adequately address the reviewers' concerns and meet the standards of the [Journal Name]. We eagerly await further guidance and feedback from you and the reviewers.

Thank you for your time and consideration.

Sincerely,

Md. Rashadul Islam
