# Supplementary material for: Smoking and pre-existing co-morbidities as risk factors for developing severity of COVID-19 infection: Evidence from a field hospital in a rural area of Bangladesh
Source: PLoS One. 2023 Dec 8;18(12):e0295040. doi: 10.1371/journal.pone.0295040 (PMC10707513; doi:10.1371/journal.pone.0295040)
Supplement: Attachment — Submitted filename: Response to Reviewers-2.docx [file pone.0295040.s005.docx]

Date: 04.11.2023

Subject: Response to Reviewers - Manuscript Number PONE-D-22-33469R1.

Dear Steve Zimmerman,

We would like to express our sincere gratitude to you and the reviewers for taking the time to assess our manuscript titled "Smoking and Pre-existing Co-morbidities as Risk Factors for Developing Severity of COVID-19 Infection: Evidence from a Field Hospital in a Rural Area of Bangladesh." We value the constructive feedback provided by the reviewers, and we have carefully addressed each point raised in the reviews.

Please find our detailed responses below, along with the corresponding changes made in the revised manuscript.

**Reviewer #2: Thank you for addressing the previous comments and the great work on the manuscript language.**

**Reviewer comment #1:** In line 97, the word "hospitalization" is duplicated, please delete.

**Author repones:** Thank you. Now the duplicate has been removed.

**Reviewer comment #2:** from line 110 to 112: please rephrase this to be more understandable. For example: It used to serve as ... and since August 27, 2020, it began to serve as .. and continue until now.

**Author response:** We appreciate your feedback. It has now been rephrased as ” *The hospital, initiated as part of COVID-19 outbreak management in Teknaf, began operations on August 27, 2020, and it is still actively providing services to the local population and Forcibly Displaced Myanmar Nationals (FDMN) as a not-for-profit organization*”

**Reviewer comment #3:** In line 116: since the hospital, it's services and supplies still continue to serve people for free, please adjust the grammar of the sentence accordingly.

**Author response:** We've corrected the grammar, and it now reads “*Besides; 24/7 Emergency, Out-Patient Department, radiology, laboratory services, and referral by ambulance to higher level facilities in Cox’s Bazar district remain available free of charge*”

**Reviewer comment #4:** In line 137: can you please clarify if this sentences is being referenced to reference number [32] and what information the reference is supporting? [32]: if not, please check your references, as reference [32] is only mentioned in this line.

According to your response to the reviewer, you stated that "We chose to categorize patients as "adults" (18-60 years) and "elderly" (>60 years) due to the observed significant association between these age groups and the severity of COVID-19 infection in the pilot analysis." I would suggest adding this information to the manuscript.

**Author response:** Thank you for pointing this out. There was an unnecessary text, and it has now been removed. Your suggestions have also been incorporated into the manuscript

**Reviewer comment #5:**  line 380: please rephase.

**Author response:** We have revised the line (new line #386), and it now reads: “*Also, it is recommended that public health initiatives prioritize extensive smoking cessation programs and awareness campaigns aimed at reducing smoking rates, particularly among individuals with pre-existing health conditions. Additionally, enhancing diagnostic infrastructure for those with comorbidities can aid in the timely identification of their health issues.*”

**Reviewer #4: Thank you for your revision. I find the manuscript much improved. I only have a few comments for the authors to address:**

**Reviewer comment #1:** Background, line 105-106: "...either cigarettes or other tobacco products..." Only cigarette smoking is addressed in the manuscript and so if other tobacco products were not considered it should be noted as a limitation or the reference to these should be removed.

**Author response:** We appreciate your attention to this matter. We have now clarified in the methodology section (line number #164) that our study considers both cigarette smoking and other tobacco product use as part of the broader category of smoking. This approach allows us to encompass a wide range of tobacco consumption habits.

**Reviewer comment #2:** Methods, line137: remove (why was this reference deleted?)

**Author response:** Thank you. Now it has been removed

**Reviewer comment #3:** Results, line 250: word choice "riskier" is awkward

**Author response:** We have changed the word "riskier" to "more prone".

**Reviewer comment #4:** Discussion: You did not analyze the effects of sex and smoking combined, and it is noted here in your manuscript, but it should be noted as a limitation as there may be an interaction effect on men being more likely to develop severe COVID due to more of them being smokers.

**Author response:** Thank you for your feedback. The prevalence of smoking, which may influence the severity of COVID-19, was not examined separately for males and females in our study. We acknowledged this limitation in the "Limitations" section (Line number #375), highlighting that we did not analyze the potential interaction effects between sex and smoking that could influence the development of severe COVID-19.

**Reviewer comment #5:** What do you make of the non-significance of the association between smoking status and critical COVID in the adjusted model?

**Author Response:** Thank you for your question. The non-significance of the "Critical" category in the adjusted model is likely influenced by the smaller sample size of critical cases compared to severe cases. Additionally, the presence of other significant risk factors, such as hypertension, COPD, and age, in the model may have minimized the influence of smoking status when it comes to critical cases.

**Reviewer comment #6:** line 301: "consistence" should be "consistent"

**Author Response #6:** Thank you. We have changed the word.

Thank you for your time and consideration.

Sincerely,

Md. Rashadul Islam
